# Balanced-armature-based, electromagnetic actuator for round window stimulation of the inner ear with static pre-load

**DOI:** 10.1007/s10544-025-00766-x

**Published:** 2025-12-29

**Authors:** Maren S. Prediger, Eileen Müller, Anatoly Glukhovskoy, Konrad Bethmann, András Bernát Berta, Marc C. Wurz, Hannes Maier

**Affiliations:** 1 Institute of Micro Production Technology, Leibniz University Hannover, Hannover, Germany; 2 Institute for Information Processing, Leibniz University Hannover, Hannover, Germany; 3 Leibniz University Hannover, Hannover, Germany; 4 Department of Otorhinolaryngology, Hannover Medical School, Hannover, Germany; 5 Cluster of Excellence “Hearing4all”, Hannover, Germany

**Keywords:** Active middle ear implant, Balanced armature, Round window stimulation, Round window actuator, Electromagnetic actuator

## Abstract

Roughly 5% of the population is affected by hearing loss. In case of a functional middle ear impairment with decreased transmission, different types of active middle ear implants may restore hearing. To drive the round window membrane (RWM) for example, bone anchored actuators or floating mass transducer (FMT) have been used in clinics. Bone anchored devices are usually too big for the round window niche and FMTs display low displacement and force at low frequencies. This paper focuses on the stimulation of the RWM by a stator-based actuator that combines small size and bone anchoring. The design features a rotational-symmetric balanced armature core with flexible membrane parts manufactured micro technologically. Membranes and assembled prototypes were evaluated on the bench in the range of 0.1 kHz – 10 kHz and prototypes tested in human temporal bones. We demonstrate the feasibility of a balanced armature actuator of appropriate size for RW placement. With 1.4 mm outer diameter and 4.9 mm length, the actuator fits the anatomical constraints. Acoustic stimulation of the RWM resulted in a maximum output of approximately 80 eq. dB SPL at 300 Hz increasing to > 100 eq. dB SPL above 5 kHz. The measured output is comparable to clinically used devices. The design lacked sufficient robustness leading to a low yield in manufacturing and failures in experiments, although the output level was insensitive to static force loading up to 30 mN. Future developments require an increased maximum output level and improved robustness.

**Graphical abstract:**

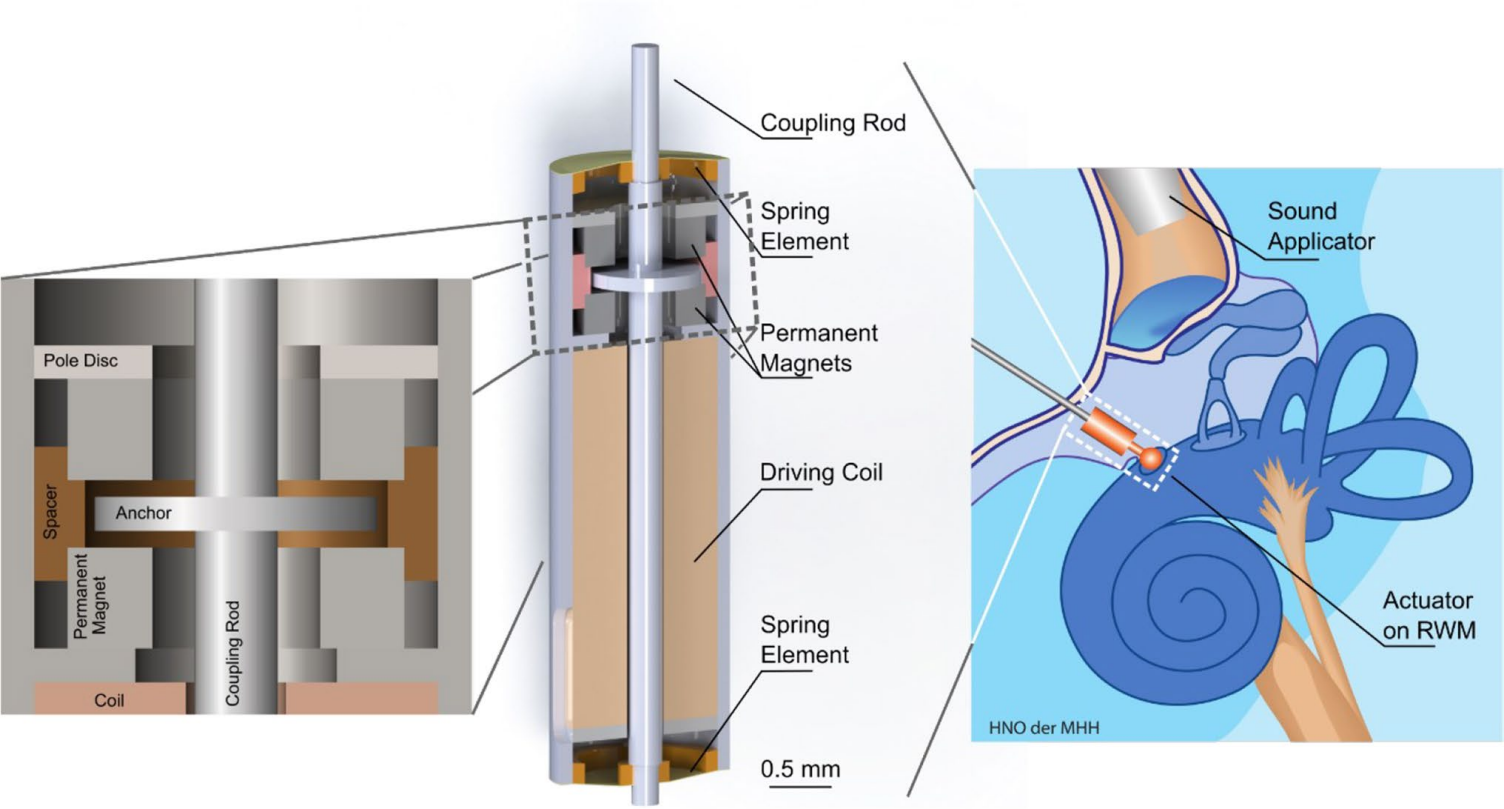

**Supplementary Information:**

The online version contains supplementary material available at 10.1007/s10544-025-00766-x.

## Introduction

Roughly 430 million people worldwide are affected by hearing loss (WHO 2024), which is divided into conductive and sensorineural hearing loss. Conductive hearing loss is caused by a decreased middle ear transmission, as found with otosclerosis (fixation of the ossicles and stapes footplate) or with loss of the ossicles due to trauma, which attenuates sound transmission to the inner ear. Initially, active middle ear implants (AMEI) were developed as an alternative to conventional hearing aids to treat sensorineural hearing loss. In 2006, Colletti et al. demonstrated clinically, that the FMT of the Vibrant Soundbridge (VSB, MEDEL, Austria) can be placed onto the RWM, successfully driving the inner ear in the reverse direction (Colletti, Carner et al. 2006). Since then, RW stimulation has grown into a successful treatment for mixed hearing loss, circumventing the transmission of sound by the middle ear (Kiefer, Arnold and Staudenmaier 2006, Wollenberg, Beltrame et al. 2007). However, there is still a high variability in patient outcomes and the output falls short of theoretical prediction in some patients (Beltrame et al. 2009). Over the following years, accessories for the VSB and other existing partially and fully implantable AMEIs were developed (Lefebvre, Martin et al. 2009, Iwasaki, Suzuki et al. 2012). Also, completely new actuator concepts that bypass and substitute the middle ear entirely by stimulating the cochlea with a piston prosthesis through the oval window (Hausler, Stieger et al. 2008). In previous studies, it was demonstrated experimentally (Salcher et al. 2014; Muller et al. 2017) (Schraven et al. 2012), that intimate contact and a static force preload to the RW improve transmission and reduce variability in coupling efficiency. In the case of the VSB, this lead to the design of a coupler that ensures static preload force and contact to the RW while keeping the FMT mobile, which was successfully translated to clinical applications (Knölke, Murawski et al. 2022). Although FMTs have the advantage that they require no external fixation and can be used in evolving structures, such as growing skulls in kids, these actuators need to be kept mobile and provide limited force output at low frequencies. For these cases, actuators that rely on a tight fixation to the skull instead of an accelerated mass as support are advantageous. In an earlier experimental study, it was shown that the balanced armature actuator of the Direct Acoustic Cochlea Stimulator Partial Implant (DACS PI, Phonak Acoustic Implants SA, Switzerland) can be used for RW stimulation (Maier et al. 2013). Although this device was not designed for static force preload and its size is not compatible with human anatomy, RW stimulation with adequate preload yielded sufficiently high output and low total harmonic distortion in experiments.

## Concept

**Fig. 1:**
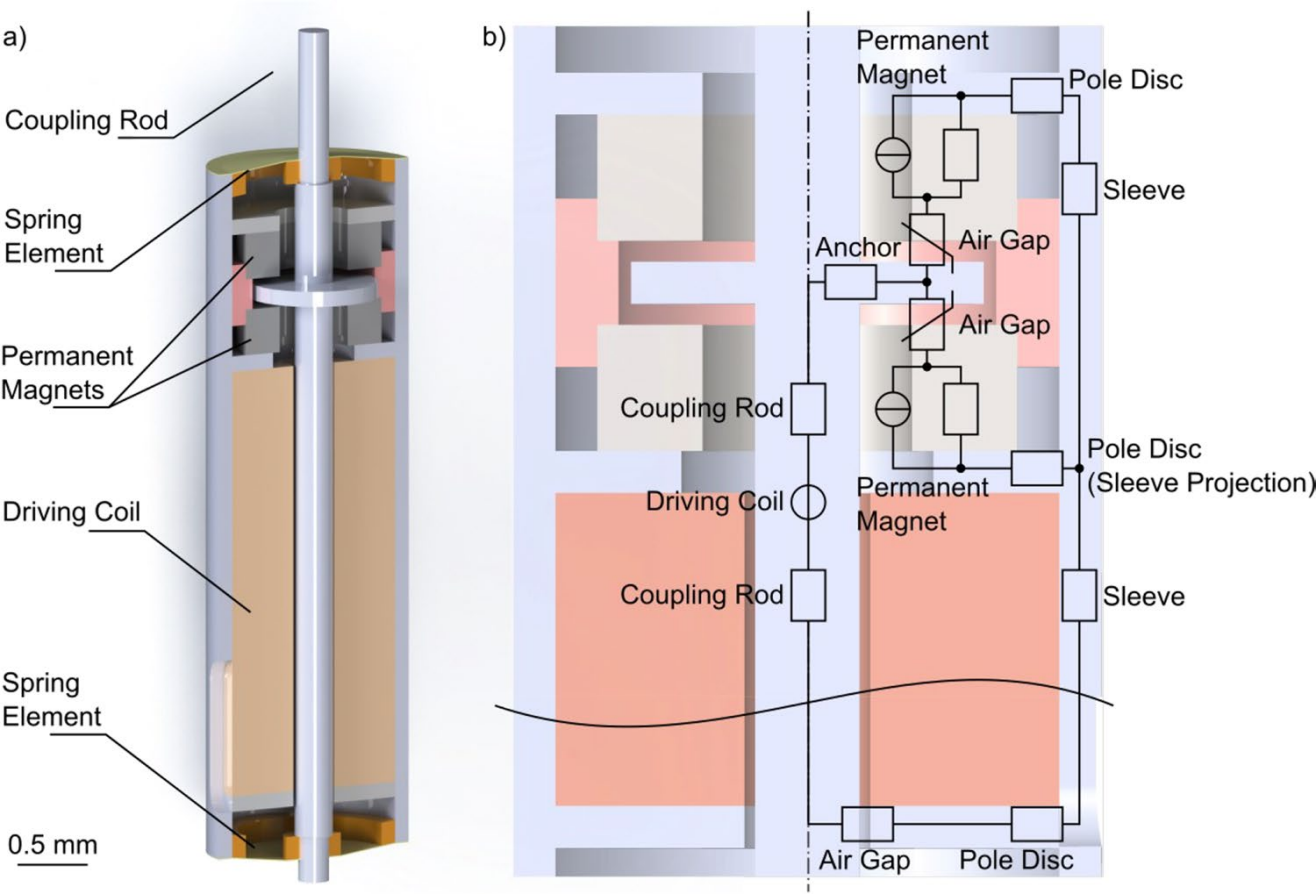
(**a**) Proposed actuator design with two main membranes at the top an bottom, acting as spring elements (labeled *Spring Element*); (**b**) Overview of the magnetic system

Consequently, the authors aimed to demonstrate feasibility with a reduced size concept for RW stimulation based on a previously published design by Cochlear Ltd. (Schreier 2012) that fulfills the necessary technical and anatomical requirements. The actuator is an electromagnetic actuator based on the balanced armature principle, where the core functional part is a magnetizable coupling rod with an integrated anchor disc balanced between two permanent magnets (Fig. 1a). In its standby (equilibrium) position, the anchor disc, ideally, is centered between the magnets and experiences equal attraction and zero net magnetic force. To drive the rod periodically, an alternating current coil magnetizes the rod so that the anchor disc will be pulled periodically toward either of the permanent magnets. Although the pulling force applied to the anchor from either magnet is compensated in the equilibrium position, the equilibrium is not stable. The closer the anchor displaces to either magnet, the higher the pulling force towards the respective magnet. This unstable behavior is also commonly known as "negative stiffness", which is impeded with an additional component, returning the anchor to the magnetic and mechanical equilibrium. In this instance, such elements can be elastic polymer membranes working as mechanical springs and the negative and positive spring constants are used to decrease the resonance frequency of the combined system. The membranes are the critical components in a balanced anchor actuator. They provide the restoring force that returns the actuator to its equilibrium position when it is subjected to an external force. It is crucial to avoid the anchor disc attaching to the permanent magnets, since the electromagnetic force required for detaching is higher than the system could provide. A restoring spring element coupled to the rod fulfills that requirement. The system’s main components thus are: the coupling rod, the magnets, the coil, and the spring element. These components are all assembled in a sleeve that is part of the magnetic circuit and seals the system to the environment. As perfectly stated by Schreier (Schreier 2012), the mechanical stiffness of the spring element and the entire system have to match the magnetic stiffness of the electromagnetic components. Additionally, all materials used are required to be biocompatible.

Based on the anatomy of the round window niche and the available space in front of the RW prototype development was conducted within the following specifications: The outer diameter ranges between 1.2 and 1.4 mm and the length of the system (sleeve length) is between two to three millimeters and, at its absolute maximum, does not exceed five millimeters. For the RW stimulation, the axial displacement of the coupling rod needs to span 2 µm—10 µm in the frequency band of 100 Hz to 10 kHz to cover the human auditory range while not operating above 1.5 Vp. The required displacement was estimated from the middle ear transfer function at low frequencies ≤ 1 kHz (Rosowski et al. 2007), assuming equal volume displacements at the stapes footplate and the RW as well as an area ratio of 3.2 mm^2^ / 0.2 mm^2^ between the stapes footplate and the actuator tip of 0.5 mm dimeter at the RW to achieve nominally 110 eq. dB SPL at 2 µm displacement. As the actuator tip does not cover the entire RW membrane, a reserve of a factor 5 was assumed to provide sufficient maximum output with 10 µm displacement for all hearing aid applications. During the hypothetical stimulation, the tip of the displacement rod is in contact with the RWM. The actuator will need to be pressed against the membrane with a certain preload of at least 10 mN, which must be considered in the design. The actuator was designed within the confines of these specifications. The outer diameter is 1.4 mm and the length of the sleeve 4.9 mm. The sleeve features a projection to allow sectional assembly of the components. The spring element consisted of two equal membranes to seal the sleeve. A non-magnetic spacer secures the air gap between the magnets. The following sections cover the simulation of the membranes, the manufacturing of the prototypes, and temporal bone experiments with one assembled prototype.

## Simulations

COMSOL Multiphysics® with the modules AC/DC and MEMS was used simulate the magnetic and mechanical requirements for the prototype design. The membranes are the critical components in a balanced anchor actuator. If the stiffness is too low, a properly assembled actuator will fail to operate because the anchor might attach to one of the permanent magnets. Then again, the anchor displacement will be critically reduced when the stiffness is too high. Therefore, it is important to predefine and estimate the required stiffness through simulation. Magnetic and mechanical forces as well as the structural and material properties of all actuator parts are non-linear, e.g., the membrane's stiffness increases under the axial load.

The AC/DC module calculates the magnetic field distribution and the generated actuation force. The model was set up considering the magnetic circuit (Fig. 1b) consisting of soft ferromagnetic elements with high permeability, a pair of axially magnetized SmCo ring magnets, and air. The circuit's permanent magnets and ferromagnetic parts (the rod, the anchor, the pole shoes, and the sleeve) were described as non-linear magnetic materials via the H-B approximation curve. The magnetic circuit consists of the stator (pole shoes, permanent magnets, sleeve) and the mobile rod with an integrated anchor separated from the ring magnets through a working air gap of 50 µm.

**Fig. 2:**
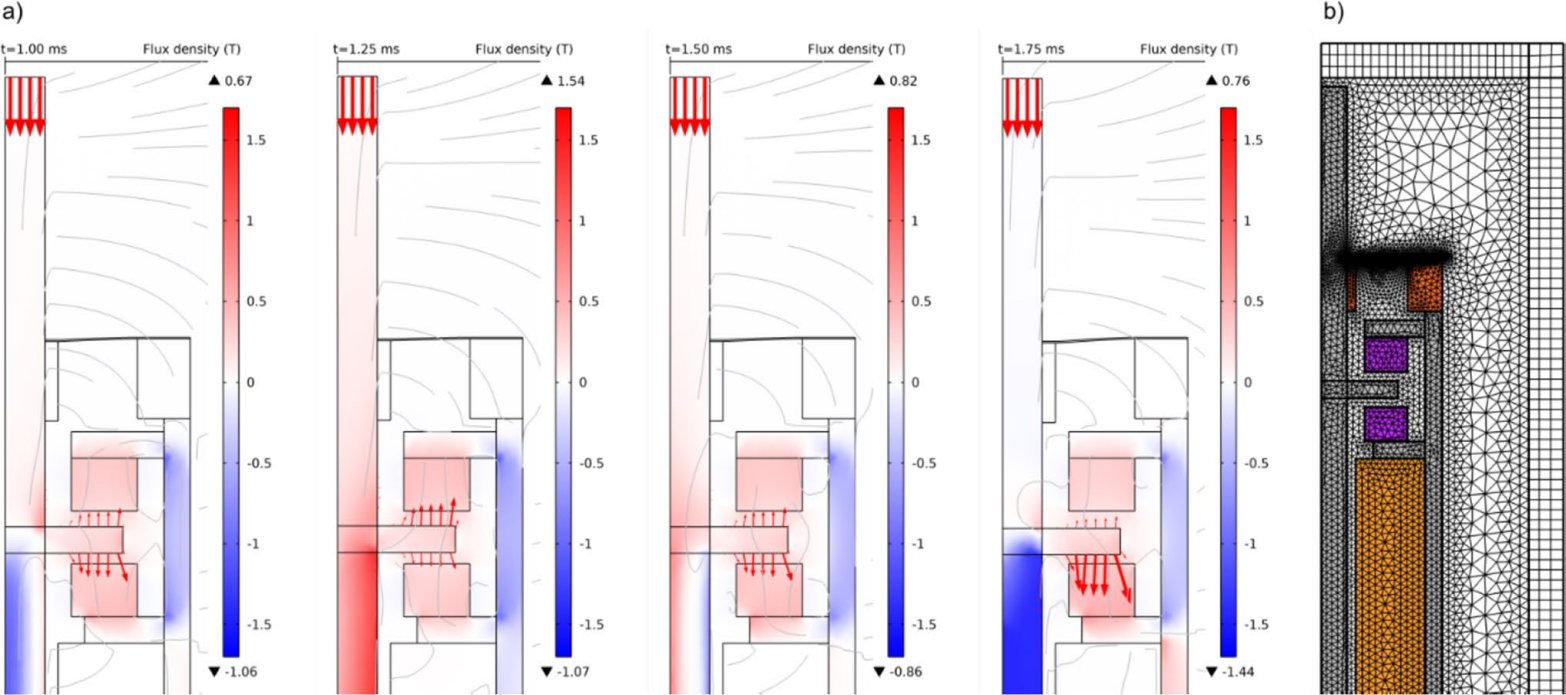
(**a**) Simulated cross-sectional view of the actuator at applied 10 mN axial load, rod position and magnetic flux density, and actuation forces shown at excitation times 1.0—1.75 ms; an excitation period equals 1 ms. Simulations show the rod in saturation at 1.25 ms and 1.75 ms. The rod being driven in saturation is the main reason for non-linear actuation and the actuation limits; **b**) cross-sectional view and mesh used for the simulation of membrane stiffness

The actual position of the anchor was determined by the MEMS module, as the resulting equilibrium of both, internal stiffness and external load. The internal stiffness is a combination of the negative magnetic stiffness and the stiffness of both membranes, the external load is the reaction force of the RWM to its displacement by the rod. To simplify parts manufacturing and facilitate the actuator's assembly, the actuator was given a cylindrical shape, as depicted in Fig. 1a. This also enables the use of an axial-symmetrical representation for the finite element analysis (FEA) model (see Fig. 2a and b), which saves computational resources and allows to solve the fully coupled non-linear model. The mechanical boundary conditions of the model were that the outer circumference of the pre-stressed membranes is constrained and the central part attached to the rod. In order to assess the influence of the axial load commonly applied in the implanted state, the actuator's performance was simulated with the actuation rod tip pressed against the RWM, with axial force ranging from 0 to 50 mN. The mechanical reaction of the RWM was set as: stiffness = 828 N/m, damping = 10^–6^ Ns/m (Heckeler and Eiber 2017). A process-determined tensile pre-stress of 25 MPa was placed on the membrane in the model. Due to the dual-membrane construction in the designs, the membranes restrict the off-axis degree of freedom and improve its robustness.

**Fig. 3:**
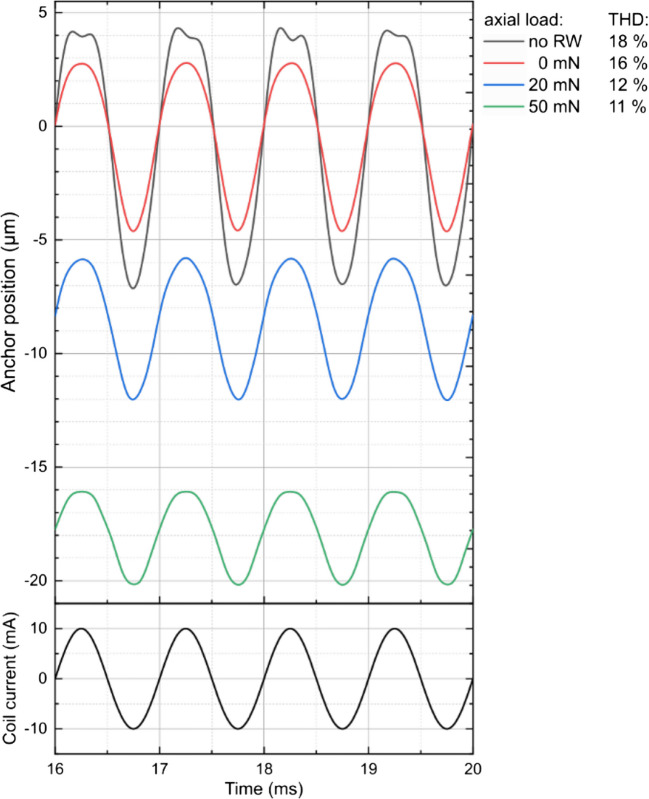
Simulated rod displacement as function of time with varying axial loads at 1 kHz and 10mAp coil excitation

Figure 3 summarizes the displacement simulations. Under the extreme load of 50 mN, the anchor was displaced 22 ± 4 µm; with a driving current of 10 mA_p_ magnitude, the working stroke was simulated to be 2 µm, which represents the smallest stimulation amplitude. The visible signal distortion originates from the magnetic saturation of the rod and the non-linear combination of the mechanical and magnetic stiffness. It was determined that the optimal total axial stiffness of the dual prestressed membrane at equilibrium is 2000 N/m.

## Components

Based on these simulations, the dimensions of all parts were set to the values listed in Table 1. The resonant frequency is expected to be between 1.5 kHz-2 kHz and 1000 N/m was defined as the goal spring constant for the individual membranes; in simulation, the stiffness was 2000 N/m for both membranes combined. SmCo was chosen for the permanent magnets, purchased from Audemars Microtec (Switzerland), which also manufactured the driving coils. For all magnetic parts, the material Permenorm© 5000 H2 was used; the material properties are listed in Table 1 (right). The sleeves and coupling rods (A) were purchased from MIKRO-PRÄZISION Wilfried Nippel GmbH (Germany). The pole discs were laser-cut in-house from 100 µm Permenorm© 5000 H2 foil procured from Sekels GmbH (Germany). The sleeves and the coupling rods underwent thermal annealing at Sekels GmbH, process proprietary. The spring element components as well as the initial spacer were manufactured in-house at the Institute of Micro Production Technology (IMPT).

**Table 1:** List of components and dimensions with tolerances

Component	Main dimensions [mm]
Sleeve	Length 4.5 Ø_OD_ 1.4 Ø_ID_ 1.2
Coupling Rod	Length 5.6 Ø_max_ 0.25 Ø_min_ 0.20
Anchor	Thickness 0.1 Ø_OD_ 0.84
Pole Disc 1	Thickness 0.1 Ø_OD_ 1.2 Ø_ID_ 0.5
Pole Disc 2	Thickness 0.1 Ø_OD_ 1.2 Ø_ID_ 0.3
Coil	Length 2.8 Ø_OD_ 1.2 Ø_ID_ 0.3
Permanent Magnets	Thickness 0.3 Ø_OD_ 1.0 Ø_ID_ 0.5
Spacer	Thickness 0.4 Ø_OD1_ 1.2 Ø_OD2_ 1.0 Ø_ID_ 0.9
Membrane on Si	Thickness 0.307 Ø_OD_ 1.4 Ø_ID_ 0.2
Membrane on PI	Thickness 0.107 Ø_OD_ 1.4 Ø_ID_ 0.2

Mechanical processing of soft magnetic material, as used here, can lead to residual stress that influences the magnetic properties and might reduce functionality. Thermal annealing can reduce these residual stresses. All magnetic components were therefore analyzed regarding their magnetic properties with a Vibrating Sample Magnetometer (Model 7407, Lake Shore Cryotronics, Inc.) before and after the thermal annealing (Sekels GmbH). The values are shown in Table 2; in general, the saturation magnetization increased for all measured parts, while the coercivity decreased. Since the actuator’s displacement is based on the saturation magnetization of the rod and the rod is driven in a state of magnetic saturation for highest displacement, the reduction of coercivity is not relevant for the application. The thermal annealing therefore caused no negative change in magnetic properties with respect to the application.

**Table 2:** Bulk material properties Permenorm © 5000 H2, based on manufacturer data sheet (Vacuumschmelze 2024)

Material Properties Permenorm© 5000 H2	
Alloy content	45–50% Nickel
Alloy structure	Isotropic, coarse-grained
Vickers hardness	220–300
µ4 (bulk)	7000
H_c_	0.05 A/cm
J_s_	1.55 T
ρ (density)	8.25 g/cm^3^
ρ (resistivity)	0.45 Ωmm^2^/m
Curie Temperature	440 °C
Young`s Modulus	140 kN/mm^2^
Coefficient of Thermal Expansion	10 × 10^–6^/K

## Spring elements

**Fig. 4:**
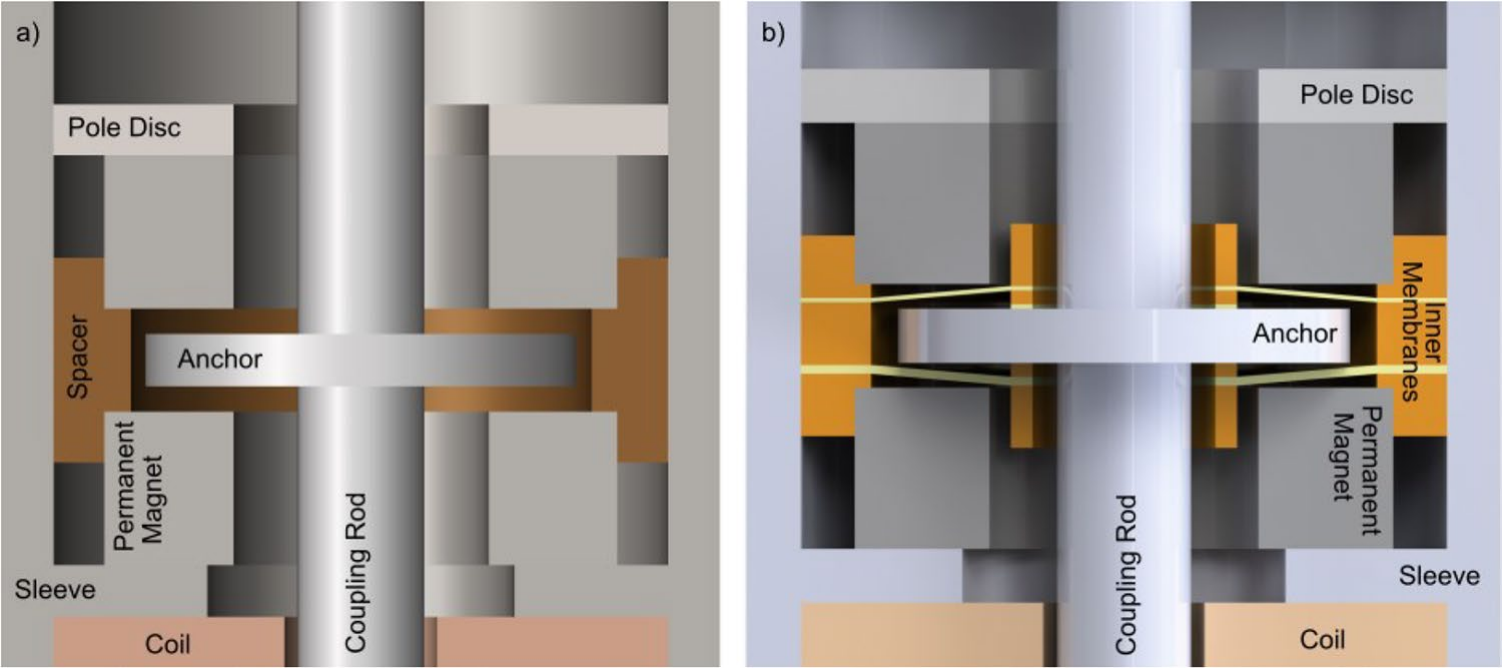
**a**) Version 1 close-up: Anchor disc between permanent magnets with a non-magnetic spacer; the membranes (*Spring Element*) are depicted in Fig. 1a, located outside of the close-up; **b**) Version 2 close-up: with inner membranes surrounding the anchor disc

The spring elements for the actuator prototype were developed in two versions. The first version consisted of two equal membranes to seal the sleeve (as seen in Fig. 1a and close-up in Fig. 4a; named outside membranes). With the coupling rod and anchor disc forming a monolithic entity and the rod having recesses at the bottom and top to interlock with the membranes, the two membranes provide axial and radial alignment, and dampen the displacement of the coupling rod. The second version features an additional set of membranes placed between the permanent magnets, which surrounds the anchor disc (named inner membranes). The placement is shown in Fig. 4b. This intends to reduce the risk of anchor attachment to the magnets, on one hand. On the other hand, the additional set of membranes was thought to increase the axial alignment and reduce non-linear rod displacement. The outer membranes require a lower stiffness, while the restoring spring force is created by the set of inner membranes.

### Version 1: outer membranes

For the first version, the outer membranes were manufactured in two ways: Non-magnetic polyimide on a silicon frame (method 1) and a membrane comprised of polyimide on a polyimide frame (method 2).

**Fig. 5:**
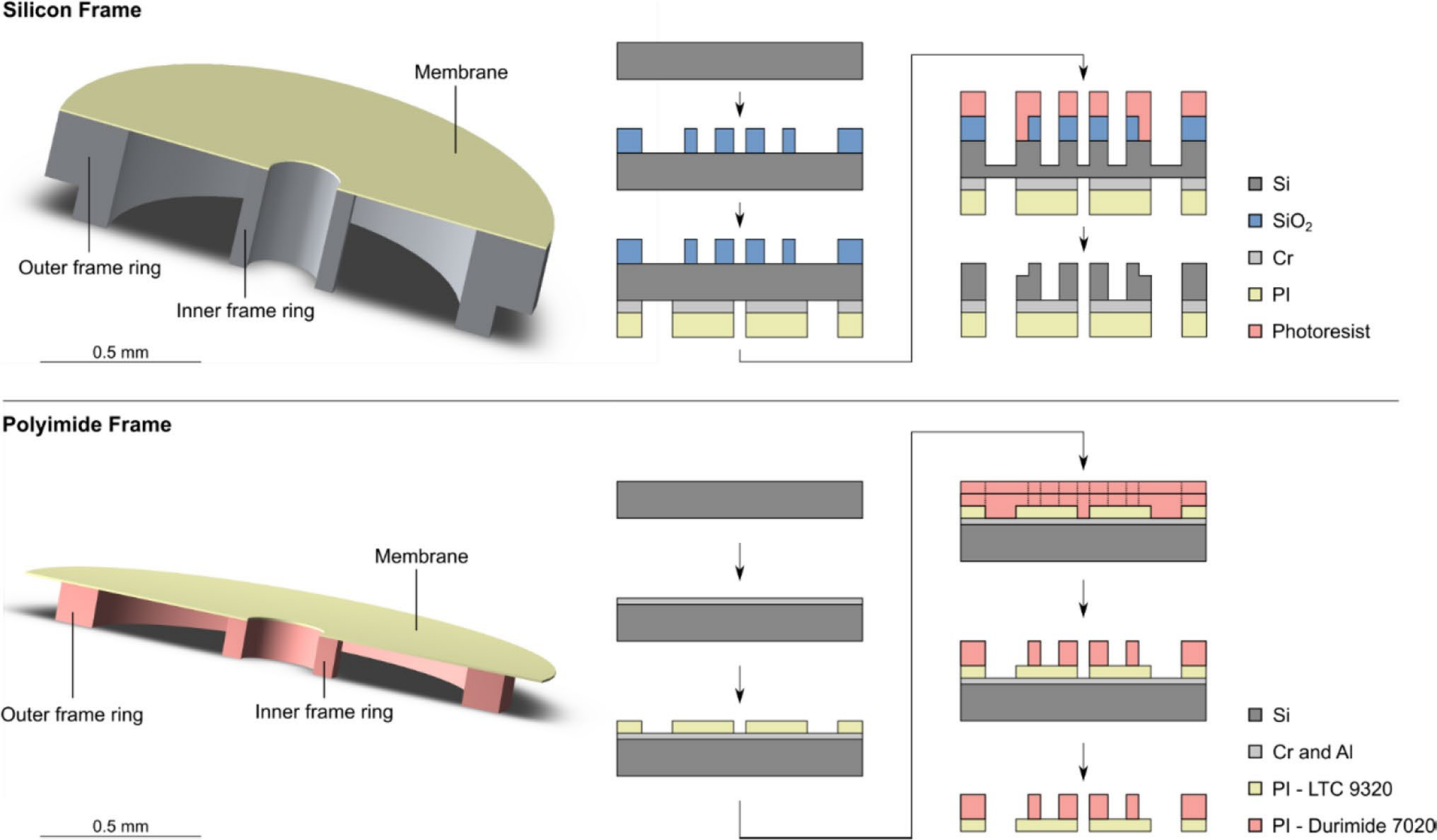
Manufacturing steps for outer membranes on a silicon frame (top) and on a polyimide frame (bottom) (Glukhovskoy et al. 2021)

The process of membrane manufacturing with method 1 (silicon frame) begins by cathode sputtering of silicon dioxide on a silicon wafer to generate a silicon dioxide mask in order to pattern the silicon wafer using deep reactive ion etching (DRIE). To create a recess in the outer frame ring (shown in Fig. 5), photoresist AZ10 XT is spin-coated on the silicon dioxide and structured with a wider diameter for the outer frame ring. On the backside of the wafer, chrome is deposited as an etch stop and polyimide LTC 9320 is spin-coated on chrome and structured photolithographically. DRIE in two steps creates a recess, which is used to center and place the frame on the rim of the sleeve.

The second method uses photolithography not just for the membrane, but also for the frame. For this purpose, a layer of chrome and aluminum is sputtered on a carrier substrate as sacrificial layer for separation. Onto the carrier, polyimide LTC 9320 is spin-coated and structured. After a thermal treatment, Durimide 7020 is spin-coated in two layers and structured to create the frame. With this method, no recess is necessary, because the outer diameter of the frame is reduced so that the frame can be placed in the sleeve and the membrane itself is placed on the rim of the sleeve. Lastly, the structured membrane is separated from the carrier substrate by anodic dissolution.

### Version 2: inner membranes

**Fig. 6:**
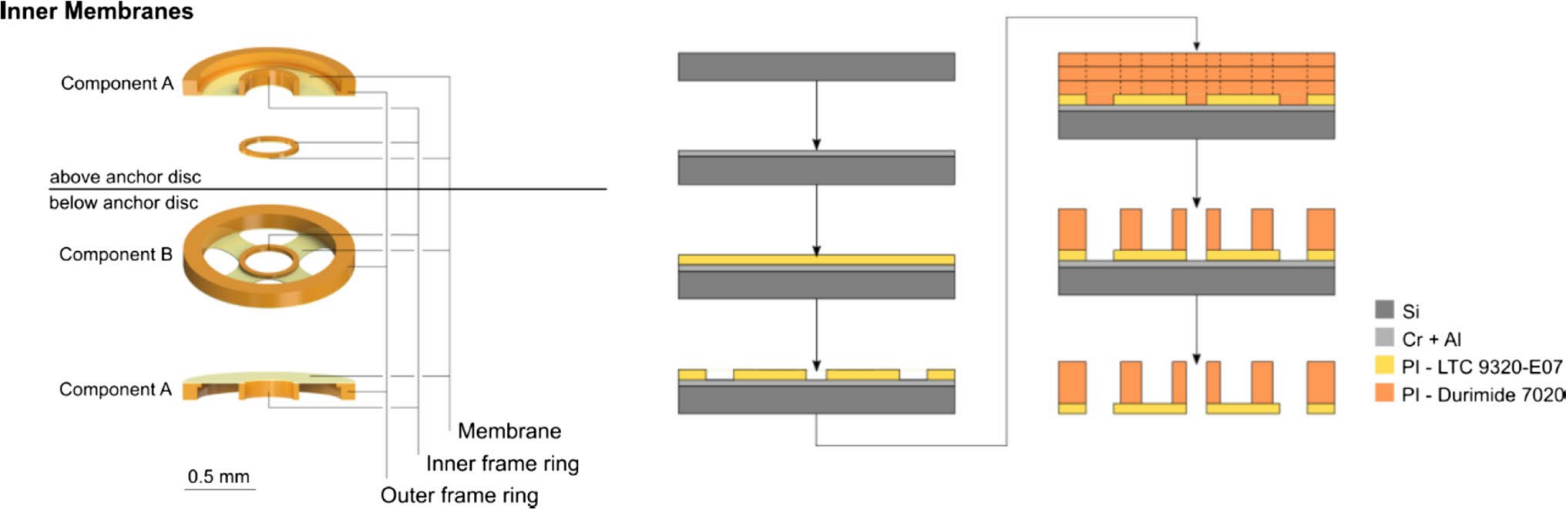
Manufacturing steps for inner membranes

The manufacturing method for the additional set of inner membranes (shown in Fig. 6 on the right) is also based on photolithography. The set is comprised of one component A plus an inner frame ring above the anchor disc and a combination of components A and B below the anchor disc. This leads to a stronger inner membrane below than above the anchor disc, which may counteract preloading against the RWM (see also Fig. 4 b). To build the inner membranes, a layer of chrome and aluminum is used as a sacrificial layer. The polyimide LTC 9320 is spin-coated on top and structured. Three layers of Durimide 7020 are used to create a total frame height of about 130 µm. The membrane was separated by anodic dissolution.

## Membrane evaluation and prototype assembly

After manufacturing, the membranes were evaluated (Table 3) regarding the resonance frequency and spring constants and prototypes assembled thereafter by laser-doppler vibrometry (OFV-552, Polytec) on a shaker. The membranes on silicon frames proved to have little adherence to the silicon. This is mainly attributed to the thermal expansion mismatch of silicon and polyimide and the shrinkage of the polyimide during heat cure. This imposes a mechanical strain toward the center of the membrane and causes the polyimide to detach from the silicon frame. The membranes on polyimide frames showed no such behavior. Where possible, the different membranes were evaluated by laser-doppler vibrometry for their resonance frequency and by nanoindentation (Hysitron TI 900, Berkovich tip) for their spring constant.

**Table 3:** Magnetic properties of the magnetic components before and after thermal annealing; magnetic properties of SmCo permanent magnets

Component	B_S_ [T]		H_C_ [kA/m]		µ_r_
Thermal Annealing	before	after	before	after	
Sleeve	1.53	1.61	0.85	0.30	
Coupling Rods	1.41	1.53	0.80	0.35	
Pole Shoes	1.34	1.74	0.90	0.05	
Permanent Magnets	0.99		-46.1, + 25.5		10.23

The outer membranes were clamped to a holder with minimal overlap. The measurement recorded membrane deflection against a frequency sweep. Mechanical behavior of the holder was subtracted from the response. The evaluation (Table 4) indicates that the actual stiffness of the polyimide membranes is slightly higher than anticipated. The membrane on polyimide frames comes closest to the simulation. The spring constant for the set of inner membranes is a combination of the individual components, which showed a fundamental resonance frequency of 24.3 kHz and 27.5 kHz which corresponds to spring constants of 540 N/m and 144 N/m for components A and B, respectively. It was not possible to measure the resonance frequency for a combined package; the overall spring constant, however, calculates to 1224 N/m. Therefore, the spring elements of version 1 with polyimide frame may provide the required restoring force to facilitate smooth actuation without unnecessary damping.

**Table 4:** Mechanical evaluation of outer and inner membranes

	Resonance Frequency	Spring constant
Simulation (for reference)		2000 N/m (total)
Outer Membrane Si frame	15.1 kHz	1332 N/m (one)
Outer Membrane PI frame	19.8 kHz	1053 N/m (one)
Inner Membranes (combined)		1224 N/m

**Fig. 7:**
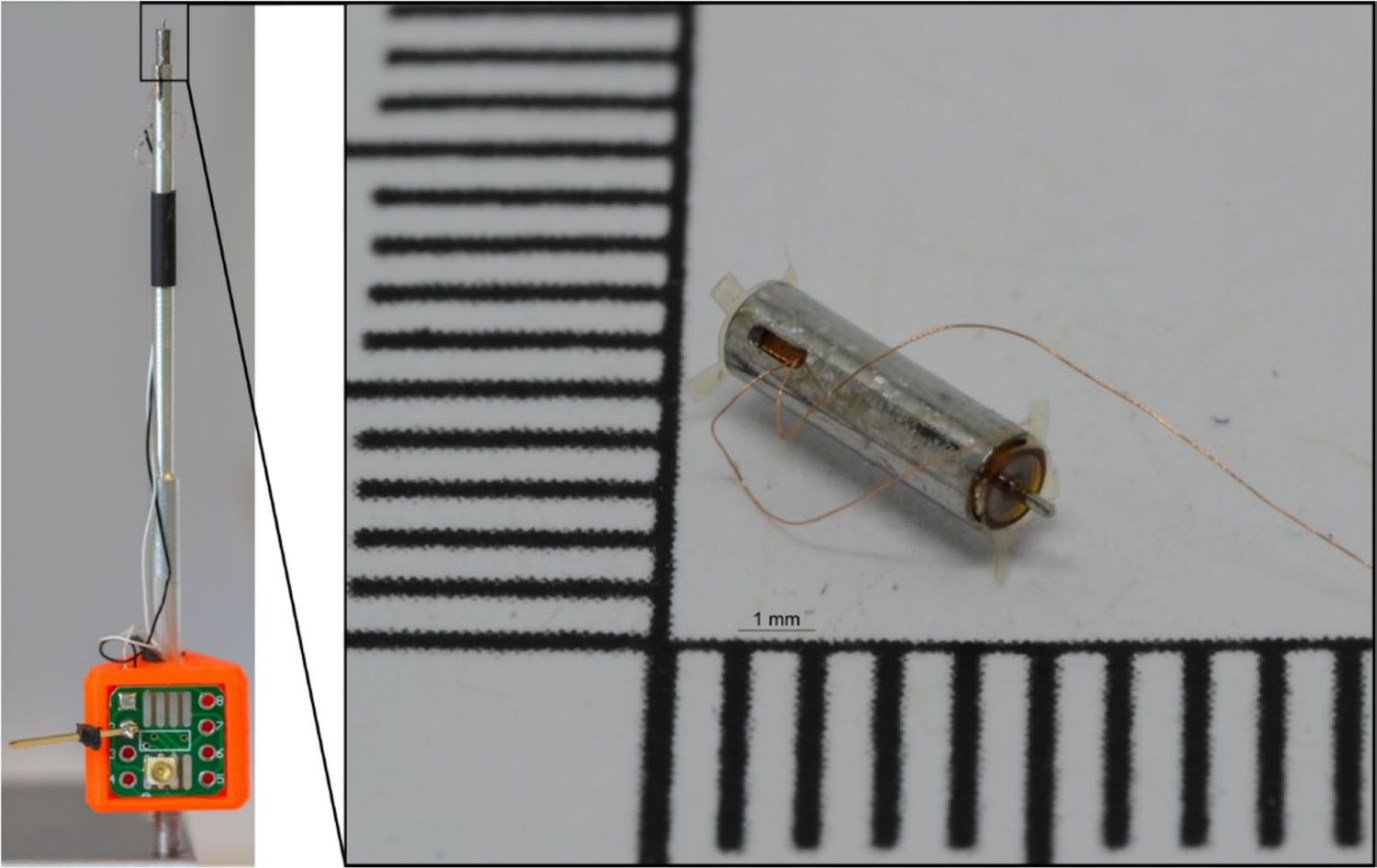
Left side: Assembled Prototype mounted on a rod and electrically connected for temporal bone experiments. Right side: Actuator with outer membranes on PI frames with PI tabs for better manual handling

The first version had the following assembly route: The coil is inserted into the sleeve and the leads thread through the slot in the sleeve. This is followed by positioning the lower pole disc and the bottom outer membrane. After that, the sleeve is turned over and the first permanent magnet is placed in the sleeve. Subsequent insertion of the spacer centers the magnet. Afterwards, the coupling rod is guided through the components to the lower membrane and positioned by the inner frame. Then the upper permanent magnet and the upper pole disc are inserted. At last, the top outside membrane is attached. Here, the inner frame ring is guided over the coupling rod so that the rod is centered by the fixation of the membrane on the sleeve. The UV curing adhesive Loctite AA 3491 was used for attachment of the different components to the sleeve. For version 2, the assembly order was adjusted: Here, after insertion of the coil and pole disc, the pole disc is fixed immediately. The lower permanent magnet is positioned and centered by the lower components of the inner membrane set. Then the coupling rod is guided through the components and the upper components of the spacer-membrane center the rod. The upper permanent magnet and the upper pole disc are inserted after that. Lastly, the lower membrane combined with a washer (surplus inner frame ring from membrane processing) and the top outer membrane is fixed to the sleeve using the UV curing adhesive. A prototype is shown in Fig. 7, with polyimide frames and mounted on a rod for temporal bone experiments.

## Temporal bone experiments

### Preparation and middle ear transfer function determination

**Fig. 8:**
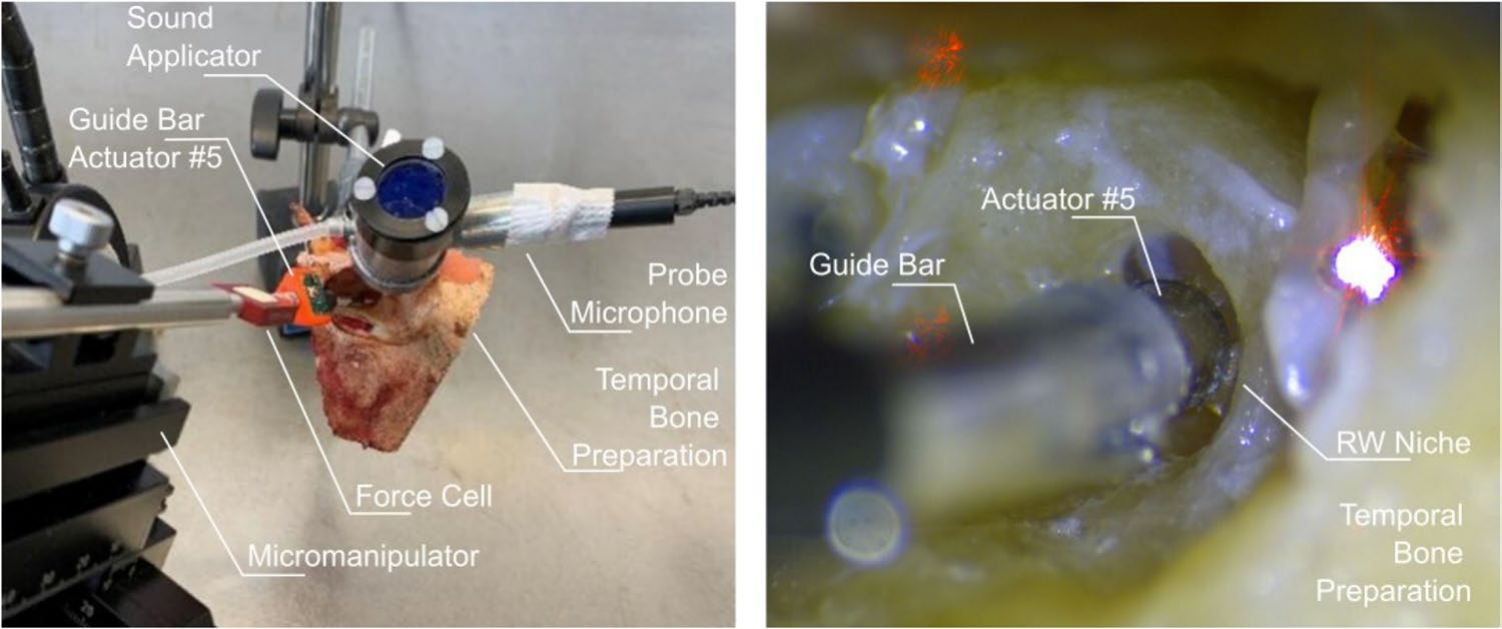
Side-by-side view of the experimental setup and the actuator placement for the determination of the middle ear transfer function and actuator stimulation of the round window

For experiments in temporal bones (TBs), an ethics committee approval had been obtained prior from the local ethics committee at Hannover Medical School (EC approval No. 9420_B0_K_2020). Human cadaveric TBs were harvested within 48 h *post mortem*, immediately frozen at -19 °C, thawed at room temperature and used within 8–12 h for the experiments. After a mastoidectomy, the facial nerve was dissected and the RW niche overhang was removed, leaving approx. 0.5 mm to 1 mm of the rim. During experiments, the TBs were periodically moistened with saline. The middle ear transfer function of the TB preparations in response to sound was measured to confirm their normal functionality according to the ASTM standard 2504–05 (ASTM International 2005). During experiments, TBs were held in a laboratory clamp positioned on a vibration isolated table (LW3048B, Newport, Germany). A custom-made sound applicator containing a probe microphone (ER-7C, Etymotic Research Inc., USA) and a loudspeaker sound supply (DT 48, beyerdynamic, Germany) was cemented (Paladur, Heraeus Kuzler GmbH, Germany) into the outer ear canal. The probe microphone was positioned 1 mm – 2 mm in front of the tympanic membrane (TM). Stapes vibration in response to sound (~ 90 – 105 dB SPL) and to actuator stimulation was measured on the posterior crus using Laser Doppler vibrometry (LDV, HLV 1000, HLV MM2, Polytec, Germany). To increase the reflectance, a small piece (0.3 mm x 0.3 mm) of retroreflective tape (Polytec, Germany) was placed at the measurement site. The visually estimated angle of incidence of the laser beam was 30° to the normal of the stapes footplate and was considered during analysis by a cosine correction of the vibration magnitudes. The setup is shown in Fig. 8.

### Signal generation and acquisition

In all experiments, the input voltage to the actuator was 1.5 Vp. A 24-bit USB data acquisition card (USB-4481, National Instruments, USA) in combination with a custom-written program (LabView, Version 15.0.1) was used to generate the input voltage for the actuators and to record the output signal of the LDV system. The loudspeaker and actuators were electrically driven by a sequence of 23 sine wave signals with stimulation frequencies in the range between 0.1 kHz to 10 kHz. Responses were averaged 30 – 500 times until a signal-to-noise ratio (SNR) of 12 dB or the maximum number of averages was reached. Only results with a SNR higher than 12 dB contributed to analysis.

### Actuator functionality test

**Fig. 9:**
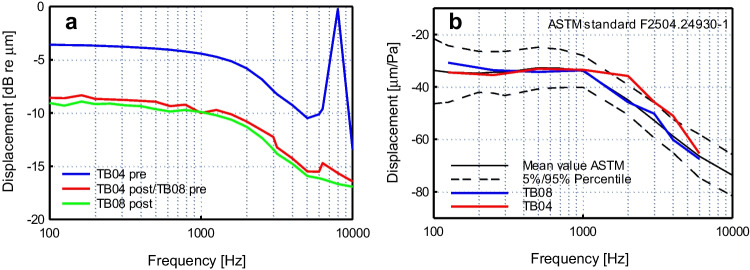
(**a**) Controls of unloaded displacement output of actuator #5 before and after experiment in TB04 and the subsequent experiment in TB08 when driven with an input voltage of 1.5 Vp. (**b**) Middle ear transfer function of temporal bones TB04 and TB08 before experiments. Dashed lines depict the 5%/95% percentile range of the extended ASTM range (Rosowski et al. 2007)

Five actuators were available for functionality tests. In order to test the functionality of the actuators, the displacement of the actuators was measured on the bench using LDV with the laser of the LDV focused in axial direction onto the tip of the unloaded actuator. Four actuators showed a displacement amplitude < — 40 dB re 1 µm. Only one actuator (#5, Fig. 9a) displayed deflection in the range of -4 to -13 dB re 1 µm and was therefore used for TB experiments.

### Actuator stimulation in temporal bones

To determine the equivalent sound pressure level (eq. dB SPL) generated by the actuator, the cochlea was first stimulated acoustically by the loudspeaker and secondly by the actuator at the round window membrane with different preloads. The displacement response to both stimulation modes of the stapes footplate was measured using LDV. During stimulation, the actuator tip was pressed against the RWM with a defined static force determined by a force cell with a force range ± 1 N (FUTEK USB210, USA) and adjusted using a micromanipulator. Response to actuator stimulation at an input voltage of 1 V_RMS_ was measured on the posterior crus. Subsequently, the equivalent SPL was calculated according to the ASTM F2504-05 Standard Practice for Describing System Output of Implantable Middle Ear Hearing Devices (ASTM International 2005; Rosowski et al. 2007).

## Results

### Functionality test

Before and after temporal bone experiments, the unloaded output displacement of the actuator was tested for its performance by LDV to verify stability of the actuator output (Fig. 9a). After the first experiment (TB04), with static force loads up to 30 mN, a drop in displacement of approximately 5 dB was apparent but remained stable in the second experiment (TB08) where forces were limited < 15mN (Fig. 9a).

### Temporal bone

To determine the equivalent sound pressure level output of actuator #5, experiments were performed in two temporal bones (TB04 and TB08) that met the ASTM acceptance criterion at all frequencies (TB08) or were close to it (TB04, except at 2 kHz and 4 kHz, see Fig. 9b).

**Fig. 10:**
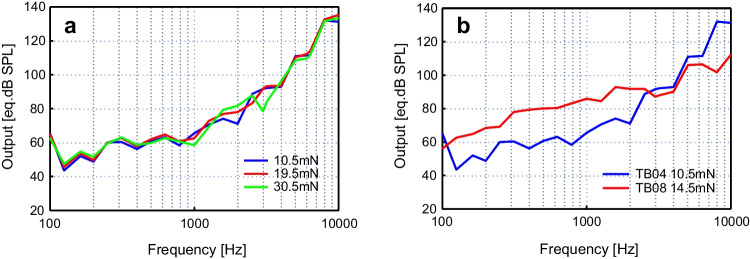
(**a**) Output of actuator #5 driving the round window in TB04 at three different static preload forces between approximately 10—30 mN when driven with an input voltage of 1.5 Vp. (**b**) Equivalent sound pressure output of actuator #5 with an input voltage of 1.5 Vp driving the round window in temporal bones TB04 (red) and TB08 (blue)

Figure 10 depicts the equivalent sound pressure level output generated by actuator #5 in temporal bones TB04 and TB08 when coupled to the RWM. In TB08, the output level was determined with a static preload of 14.5 mN, whereas in TB04, preload forces of 10.5 mN, 19.5 mN and 30.5 mN were investigated. In TB04, no significant influence of the contact pressure was observable (Fig. 10a). Although pre-post controls of the unloaded actuator displacement output on the bench showed that the baseline output of the actuator decreased by ~ 5 dB during the first experiment (Fig. 9a), the measured output was ~ 20 dB higher in TB08 at a comparable static force (Fig. 10b). In TB08 the obtained output sound pressure level was ~ 80 eq. dB SPL or higher at frequencies > 300 Hz.

## Discussion

AMEIs are a successful treatment for mixed hearing loss, although high variability in patient outcomes still poses a challenge. It was demonstrated (Schraven et al. 2012; Salcher et al. 2014; Muller et al. 2017), that intimate contact and a static force preload to the RW improve transmission and reduce variability in coupling efficiency. FMTs have the advantage that they require no external fixation, but rely on an accelerated mass as support, provide limited force output at low frequencies. In an earlier experimental study, we showed that an balanced armature actuator can be effectively used for RW stimulation (Maier et al. 2013). However, as the device used in this study is not compatible with human anatomy, our intention here was to design a miniaturized balance armature actuator and to demonstrate the feasibility of RW stimulation with adequate preload and sufficiently high output experimentally. Consequently, the authors aimed to demonstrate feasibility with a reduced size concept of 1.4 mm outer diameter and 4.9 mm length for RW stimulation based on a balanced armature principle with a modified magnetic/mechanical equilibrium for moderate static load.

In a first prototype with polyimide membranes on silicon frames as spring/housing elements, the membranes detached from the silicon frame, probably due to a thermal expansion mismatch of silicon and polyimide. Hence, a second prototype with a polyimide membrane on a Durimide 7020 frame was designed and manufactured that was stable and used for testing in human temporal bones.

Testing the unloaded actuators on the bench demonstrated that the design specifications of 2 µm to 10 µm displacement output in axial direction were not reached by a factor of approximately -20 dB less. The driving input was 1.5 Vp, a typically voltage available in hearing aids. In addition, the actuator showed a decline in output performance after the first experiment in human TBs resulting in an even lower displacement output of -10 to -15 dB re 1 µm in the second experiment.

Despite these obvious drawbacks, the actuator demonstrates the feasibility of several important aspects: (1) the output displacement achieved in the second experiment was ~ 80 eq. dB SPL or greater at frequencies > 300 Hz. This covers the relevant frequency range for human communication between 0.5 – 4.0 kHz (ANSI 1997), but remains below the intended specifications. Nevertheless, this measured output is in the order of currently clinically used active middle ear implants in RW application, even if the reserve to compensate for variability might be low (Maier et al. 2024). (2) Although the balanced armature design requires a sophisticated balance between elastic forces and magnetic forces, it proves possible to stimulate the RW with constant displacement output and a static force load up to ~ 30 mN. (3) Most importantly, the former aspects emphasizes that a balanced armature actuator, of sufficiently small size for placement and fixation in the RW niche, can be successfully micro-manufactured, even though assembly proved challenging, with 1 functional actuator out of 5 which could be used for experiments. Further, this actuator encountered a performance drop of unknown origin in the first experiment indicating a pronounced fragility during handling. The pre and post unloaded control measurement in the second experiment showed a stable mechanical behavior, more experiments, however, were not possible, as the pre-measurement before a third experiment showed a failure of the actuator. All this taken into account, it is apparent that the robustness of the current design is insufficient, but functionality was shown. As this study was an attempt to build and demonstrate the functionality of a small stator-based round window actuator, it can be seen as a first successful step.

Moving forward, several practical problems remain. Firstly, an increased robustness of the design to reduce failures and improve manufacturing yield will be required. Secondly, the achievable displacement output will have to be increased by approximately 20 dB. Sufficient maximum output will be required not only for severe sensorineural hearing loss patients by providing more dynamic range, but also to compensate for the known variability in coupling efficiency in round window stimulation (Beltrame et al. 2009). One possible source of insufficient output level in our experiments is the size of the contact element to the round window that was not explored in detail. A larger contact area compared to the approx. 1.5 mm^2^ in the current design here might be beneficial for output to the cochlea. Moreover, although polyimide is potentially biocompatible, it is not clear if it can provide long-term hermetical sealing. Current bellow designs are still larger than the here intended size (Shin et al. 2024), however this concept is promising at the mechanical output side, if smaller diameters become available and considered in the overall mechanical properties. In contrast, sealing and integration of electrical feedthroughs at the fixation end creates no obvious problems.

## Conclusion

In conclusion, we demonstrated the feasibility to manufacture a balanced armature electromagnetic actuator of appropriate size for the round window placement. With 1.4 mm outer diameter and 4.9 mm length, the actuator fits into the human middle ear round window niche. Acoustic stimulation of the round window membrane was possible with a maximum output of approximately 80 eq. dB SPL at 300 Hz increasing to > 100 eq. dB SPL above 5 kHz at a voltage commonly available in hearing aids. Although comparable in output to clinically used devices, our achieved output remained below our initial specifications. Further, the design lacked sufficient robustness leading to a low yield in manufacturing and few actuators available for experiments. This was also emphasized by an initial drop in displacement output in a first experiment, although the output was insensitive to static force loading up to 30 mN, and remained stable in the second experiment. Besides hermetical sealing, future developments will require an improved maximum output, that might be at least partially achieved by a more appropriate contact surface of the transmission rod to the RWM, and an adjustment or measurement implementation of the static contact force.

## Supplementary Information

Below is the link to the electronic supplementary material.

Supplementary file1 (GIF 3147 KB)

## Data Availability

Data sets generated during the current study are available from the corresponding author on reasonable request.
