# Supplementary figures and images for: Balanced-armature-based, electromagnetic actuator for round window stimulation of the inner ear with static pre-load
Source: Biomed Microdevices. 2025 Dec 29;28(1):1. doi: 10.1007/s10544-025-00766-x (PMC12748139; doi:10.1007/s10544-025-00766-x)

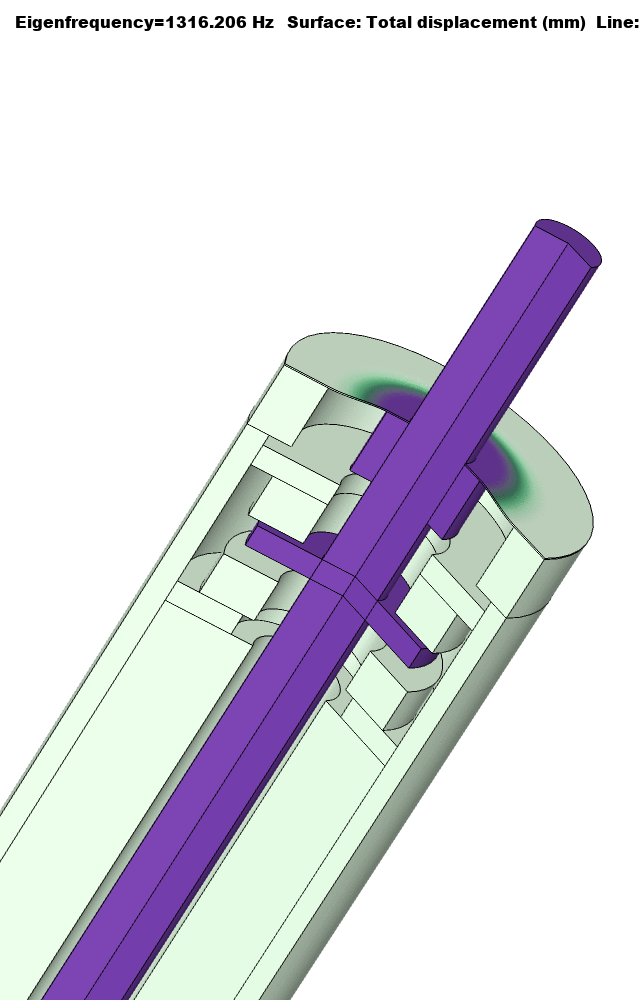

Supplement: Supplementary file 1 — Supplementary file1 (GIF 3147 KB) [file 10544_2025_766_MOESM1_ESM.gif]
